# A proteomic meta-analysis refinement of plasma extracellular vesicles

**DOI:** 10.1038/s41597-023-02748-1

**Published:** 2023-11-28

**Authors:** Milene C. Vallejo, Soumyadeep Sarkar, Emily C. Elliott, Hayden R. Henry, Samantha M. Powell, Ivo Diaz Ludovico, Youngki You, Fei Huang, Samuel H. Payne, Sasanka Ramanadham, Emily K. Sims, Thomas O. Metz, Raghavendra G. Mirmira, Ernesto S. Nakayasu

**Affiliations:** 1 Department of Biology, Brigham Young University, Provo, UT 84602 USA; 2 Biological Sciences Division, Pacific Northwest National Laboratory, Richland, WA 99352 USA; 3 Department of Medicine, The University of Chicago, Chicago, IL 60637 USA; 4 Department of Cell, Developmental, and Integrative Biology, and Comprehensive Diabetes Center, University of Alabama at Birmingham, Birmingham, AL 35294 USA; 5 Department of Pediatrics, Center for Diabetes and Metabolic Diseases, Indiana University School of Medicine, Indianapolis, IN 46202 USA

**Keywords:** Proteomics, Membrane trafficking, Biomarkers

## Abstract

Extracellular vesicles play major roles in cell-to-cell communication and are excellent biomarker candidates. However, studying plasma extracellular vesicles is challenging due to contaminants. Here, we performed a proteomics meta-analysis of public data to refine the plasma EV composition by separating EV proteins and contaminants into different clusters. We obtained two clusters with a total of 1717 proteins that were depleted of known contaminants and enriched in EV markers with independently validated 71% true-positive. These clusters had 133 clusters of differentiation (CD) antigens and were enriched with proteins from cell-to-cell communication and signaling. We compared our data with the proteins deposited in PeptideAtlas, making our refined EV protein list a resource for mechanistic and biomarker studies. As a use case example for this resource, we validated the type 1 diabetes biomarker proplatelet basic protein in EVs and showed that it regulates apoptosis of β cells and macrophages, two key players in the disease development. Our approach provides a refinement of the EV composition and a resource for the scientific community.

## Introduction

Extracellular vesicles (EVs) are membrane bilayer-bound particles containing lipids, proteins, nucleic acids, and saccharides that are secreted by cells^1^. EVs are mainly classified as exosomes or ectosomes depending on their biogenesis^2^. Exosomes range from 30 to 200 nm in diameter and are formed via the endocytic pathway, leading to the formation of multivesicular bodies, which are then fused to the plasma membrane and released as EVs^2^. Ectosomes represent a variety of EV types, including microvesicles and apoptotic bodies, that buds directly from the plasma membrane^2^. Microvesicles are EVs of 100 to 1000 nm in diameter, whereas apoptotic bodies are larger EVs (>1000 nm) that are formed by blebbing of cells undergoing apoptosis^1–4^. EVs have immense potential as biomarkers, as they can carry signatures of the tissues of origin and processes affected by disease^4^. However, a main challenge to studying EV function and its potential as disease biomarkers is obtaining pure preparations of EVs from biofluids. This is due to the co-presence of high amounts of contaminants such as lipoproteins and albumin^5,6^, which share some physicochemical properties^7,8^.

Several analytical techniques have been developed for the isolation of EVs, including ultracentrifugation (UC), density gradient ultracentrifugation (DGUC), cushion ultracentrifugation (CUC), polymer-based precipitation (PP), size-exclusion chromatography (SEC) and immunocapture (IC)^7,8^. These techniques have individual advantages but also suffer from unique contaminant profiles^7,8^. For instance, UC co-precipitates particles of similar density, while SEC co-fractionates particles of similar sizes^7,8^. To improve the rigor of the EV preparation protocols, the International Society for Extracellular Vesicles (ISEV) developed a guideline with recommendations on experimental design and reporting results^9^, but obtaining pure EV preparation with high yields is still a challenge. Sequential purification steps have also been explored, but they can result in substantial sample losses, with EV recovery as low as 1% after two purification steps^10,11^. Furthermore, their labor intensiveness makes application to clinical biomarker studies challenging, given the need for large numbers of samples for adequate statistical power. Therefore, there is still a need to systematically evaluate different EV isolation techniques to better understand their performance and the nature of their contaminants.

Our team has been interested in understanding the roles of EVs in type 1 diabetes (T1D) development and their potential as biomarkers of the disease, as we discussed in a recent review^3^. It has been shown that EVs carry microRNAs and chemokines that can induce β-cell apoptosis^12–14^. However, more systematic studies are needed to refine the composition of EVs to facilitate the testing of individual components on T1D development.

Re-use of data deposited in public repositories allows for combined comprehensive analysis to be performed that otherwise would not be possible with data collected in a single study. This type of study is called meta-analysis and provides an opportunity to answer new questions or re-affirm/refute conclusions from previous studies^15^. In meta-analyses, studies are systematically searched in the literature and included or excluded with well-defined criteria, allowing for a combined analysis of the data from the different studies^16^. Here, we performed a meta-analysis of published proteomics data to refine the protein composition of plasma EVs. Considering that purification procedures have different performances, resulting in distinct EV/contaminant ratios^6^, we took advantage of these ratio differences to cluster EV-specific proteins separately from contaminants based on protein abundance profiles. This resulted in clusters enriched in EV markers, therefore, highly likely to be bona fide EV proteins, which were separated from well-known contaminant proteins. We performed a systematic review of the literature to validate the enrichment of EV proteins in these clusters. As a test case, we validated one of the proteins from the EV-enriched clusters as a *bona fide* EV protein using an imaging method, which is not prone to false-positive results due to contaminants in the sample. We also compared the clusters enriched with EV proteins to study their functions and their potential as biomarker candidates for T1D. We demonstrate that the meta-analysis is a powerful approach to refine EV composition and provide a better understanding of its biological roles. In addition, the refined list of plasma EV proteins represents a resource for future mechanistic and biomarker studies.

## Materials and Methods

### Datasets

**Fig. 1:**
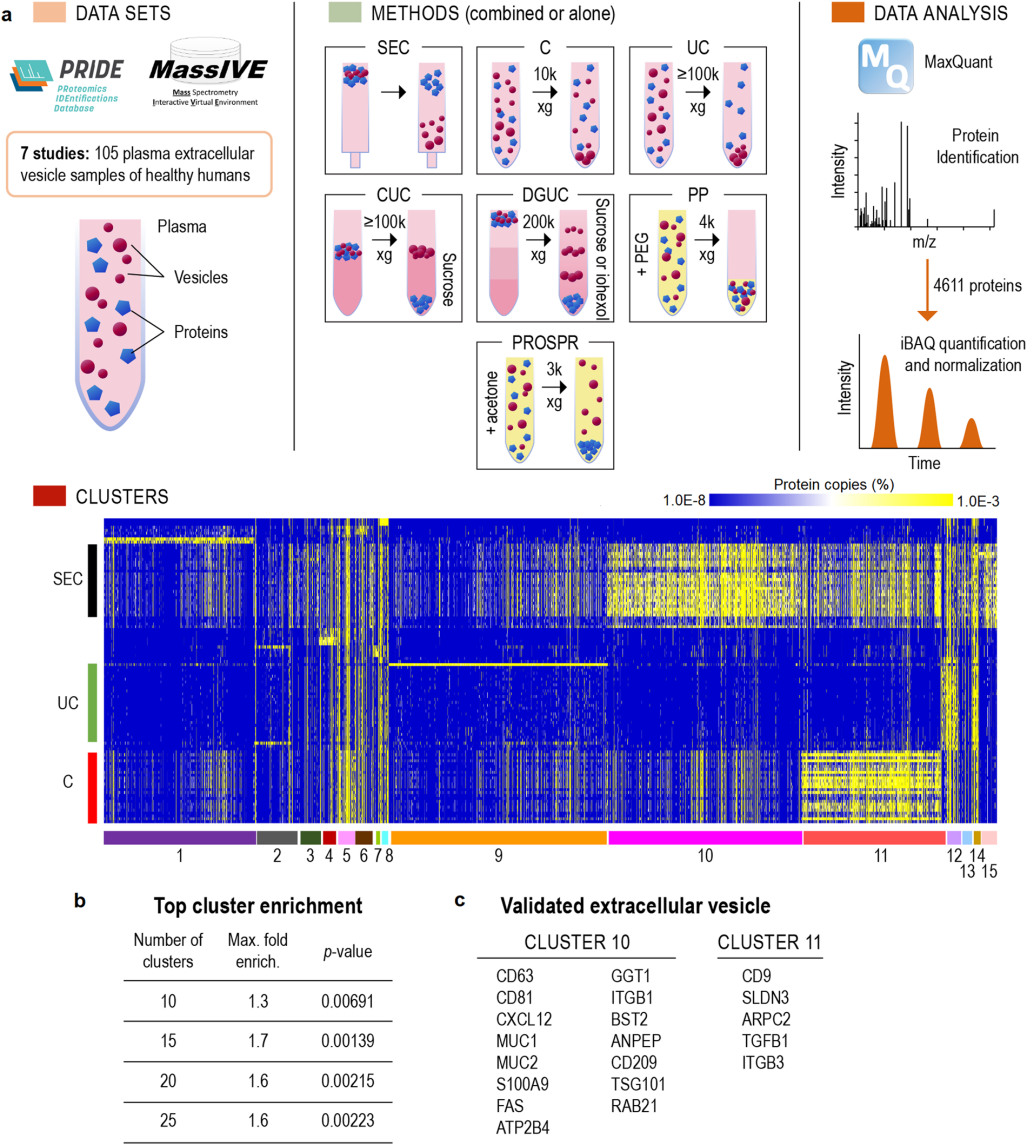
Proteomics meta-analysis of plasma extracellular vesicles (EVs). (**a**) Approaches: proteomics data from plasma EVs purified with a variety of methods were downloaded from ProteomeXchange, processed with MaxQuant and submitted to clustering analysis. Abbreviations: C - centrifugation, CUC - cushion ultracentrifugation, DGUC - density gradient ultracentrifugation, DUC - dilution followed by ultracentrifugation, PP - polymer-based precipitation, PROSPR - PRotein Organic Solvent PRecipitation, SEC - size-exclusion chromatography, UC - ultracentrifugation. (**b**) Highest enriched cluster with the top 100 extracellular vesicle proteins from Vesiclepedia when testing different numbers of clusters. P-values were calculated by Fisher’s exact test based on the distribution of expected vs. detected proteins from the top 100 Vesiclepedia proteins in each cluster. (**c**) Classical EV markers found in clusters 10 and 11.

Mass spectrometry proteomics datasets of human plasma EVs were searched in PRIDE^17^ and MassIVE^18^, the main data repositories for untargeted proteomics of the ProteomeXchange consortium. The searches were done in November and December 2021, using the keywords “extracellular vesicles”, “exosomes”, “microvesicles”, “plasma” and “serum”, following the PRISMA guidelines for meta-analysis^16^. Only label-free proteomics data collected on Thermo Orbitrap instruments with data-dependent acquisition were used (Fig. 1) to allow a consistent data analysis workflow. Isobaric tag labeling and low-resolution ion trap data would not allow to quantify the data intensity-based absolute quantification (iBAQ) method^19^, which we used for quantification. To reduce potential variability due to pathogenesis processes, only data from extracellular vesicles isolated from the plasma of healthy humans were used in our analysis. Study inclusion and exclusion criteria are listed in Fig. S1. The datasets, along with their ProteomeXchange identifiers, publications, and relevant details of the experimental methods, are listed in Table 1.

**Table 1:** Characteristics of the proteomics datasets used in the meta-analysis.

Dataset	Method	N	Purification concept	Protocol
Purification of human plasma exosomes for proteomics: optimization and application to detect changes in response to exercise^40,41^	Size exclusion chromatography (SEC)	29	Large particles elute faster in the chromatography since they are less retained by matrix.	-Removed cell debris (2,000 × *g*) and large vesicles (10,000 × *g*) by centrifugation, for 10 min each -Separated EVs by SEC using gravity columns
Plasma-derived exosomes from healthy and osteosarcoma^42,43^	Dilution followed by ultracentrifugation (DUC)	3	Dilute sample to reduce viscosity and centrifuged at lower speed removed cell debris, while EVs were recovered in main centrifugation.	-Diluted sample 8x in PBS and centrifuged at 10,000 × *g* for 30 min to remove debris -Recovered EVs by centrifugation at 110,000 × *g* -Wash pellet with PBS/centrifugation at 110,000 × *g*
Human plasma extracellular vesicles LC-MS/MS^44,45^	Centrifugation (C)	25	Centrifugation at lower speed removed cell debris and recover EVs with higher speed.	-Removed cell debris by centrifuging 2,000 × *g* for 30 min -Recovered EVs by centrifuging at 10,000 × *g* for 30 min
Tissue- and plasma-derived exosomal protein biomarkers define multiple human cancers^46,47^	Ultracentrifugation (UC)	32	Centrifugation at lower speed removed cell debris and large EVs. EVs were recovered by higher speed centrifugation.	-Removed cell debris and large EVs by centrifugation at 500 × *g* for 10 min, 3,000 × *g* for 20 min and 12,000 × *g* for 20 min -Recovered EVs by centrifugation at 100,000 × *g* for 70 min -Washed with PBS/centrifugation at 100,000 × *g* for 70 min
A novel and simple strategy to isolate extracellular vesicles from human plasma and tissue culture medium with high yield and purity^48,49^	Polymer precipitation (PP)/density gradient ultracentrifugation (DGUC)	3	The polymer precipitation binds to EVs based on their affinity to lipids, and then are separate iohexol gradient by their density.	-Added equal volume of 20% w/v polyethylene glycol PEG6000 -Centrifuged for 15 min at 4,000 × g to recover extracellular vesicles -Centrifuged on a 0–50% iohexol gradient at 200,000 × *g* for 65 h
A novel and simple strategy to isolate extracellular vesicles from human plasma and tissue culture medium with high yield and purity^48,49^	PP/SEC	3	EVs are captured by polymer precipitation followed by separation in SEC.	-Added equal volume of 20% w/v polyethylene glycol PEG6000 -Centrifuged for 15 min at 4,000 × *g* to recover extracellular vesicles -Separated by SEC
Proteome profiling of blood plasma-derived exosomes in chronic lymphocytic leukemia^50,51^	Cushion ultracentrifugation (CUC_1)	4	EVs are captured by DUC and separated on a sucrose cushion. EVs stay in the interphase, while contaminants spread into both layers.	-Removed cell debris and large EVs by centrifugation at 300 × *g* for 10 min and at 10,000 × *g* for 10 min -Recovered EVs by centrifugation at 100,000 × *g* for 100 min -Centrifugation on 40% sucrose cushion at 100,000 × *g* for 120 min -Wash EVs with PBS/centrifugation at 100,000 × *g* for 120 min
Isolation of extracellular vesicles by PROSPR^52,53^	PRotein Organic Solvent PRecipitation (PROSPR)	3	Soluble proteins are precipitated with acetone, while EVs remains in the supernatant.	-Added 4 volumes of −20 °C acetone -Vortexed for a few seconds -Removed soluble proteins by pelleting at 3,000 × *g* for 1 min
Isolation of extracellular vesicles by PROSPR^52,53^	Cushion ultracentrifugation (CUC_2)	3	EVs are captured by DUC followed by centrifugation on a sucrose cushion.	-Removed cell debris by centrifuging 300 × *g* for 30 min -Recovered EVs by centrifuging at 16,500 × *g* for 30 min -Sucrose cushion centrifugation at 200,000 × *g* for 2 h and 16 h

### Data processing

Data were processed with MaxQuant software (v.1.6.14)^20^ by matching against the human reference proteome database from UniProt Knowledgebase (downloaded on November 27, 2021). Searching parameters included protein N-terminal acetylation and oxidation of methionine as variable modifications, and carbamidomethylation of cysteine residues as fixed modification when appropriate. Mass shift tolerance was used as the default setting of the software: 20 ppm for the first search and 4.5 ppm for the second round. Only fully tryptic-digested peptides were considered, allowing up to two missed cleaved sites per peptide. Each data set was filtered with a 1% false-discovery rate at both peptide-spectrum and protein levels, resulting in a 2% false-discovery rate when all datasets were combined. Quantification of proteins was done using intensity-based absolute quantification iBAQ values extracted with MaxQuant. These values were further normalized by the total intensity of the whole sample to calculate the relative copy number of each protein for comparing proteins across different studies. The relative copy numbers were used to compare protein abundances across the different sets of data.

### Clustering and enrichment

Clustering analysis was performed using the protein abundances with MultiExperiment Viewer – MeV (v. 4.9.0)^21^. Missing values were imputed with 1/10 of the smallest value in the whole dataset to set a background level, which prevents the software to overfit the data. Clustering was done by using the k-means clustering method with Pearson correlation and a maximum of 50 iterations across all individual datasets. The k-means method was used because it separates proteins into distinct clusters. To determine the optimal number of clusters, we ran separate analyses with 10, 15, 20, and 25 clusters. The optimal number of clusters was determined by cross-checking the enrichment of the top 100 proteins that appear the most often in the literature and have been made into a resource in Vesiclepedia^22^. Enrichment was calculated based on fold-enrichment and statistical significance with Fisher’s exact test. Principal Component Analysis (PCA) was employed after eliminating proteins with missing values, resulting in the use of 51 proteins for this analysis. The goal of PCA was to reduce the data’s dimensionality and visualize the variance in the methods used for EV extraction and identify patterns and relationships among proteins. A Python script was developed to create a PCA graph, demonstrating the distribution of samples in a lower-dimensional space, and it is available on GitHub^23^ (Fig. S2).

### Systematic review of validated human EV proteins

The literature searches were done in PubMed on June 28, 2023, following the PRISMA guidelines for systematic reviews^16^. The used keywords were “extracellular vesicles”, “EV”, “microvesicles”, “MV”, “exosome” AND “immunogold” AND “human”. Studies that had no associated full text, were not conducted in human samples, were not conducted on EVs, that did not target proteins or did not use immunogold electron microscopy were excluded from the final list. The inclusion and exclusion criteria are listed in Fig. S3. Enrichment was calculated based on fold-enrichment and statistical significance with Fisher’s exact test, while the true positive rate was calculated with the following formula:

True-positive rate = (true positives)/(true positives + false negatives), being true positives the validated EV proteins that were found in EV-enriched clusters and false negatives, the validated EV proteins that were found in other clusters.

### Functional- and cell-enrichment analysis

Functional-, tissue- and cell-enrichment analyses were done with DAVID^24^ using the KEGG database with default parameters. Specific tissue markers were mapped by comparing the data against a previously published human tissue proteomics dataset^25^. Specific pathways were curated with Vanted^26^. A script was written in R to plot a bubble graph, and it is available on GitHub^23^. Networks were built and plotted with Cytoscape (v. 3.9.1)^27^. Pathway analysis was also conducted with Ingenuity Pathway Analysis (Qiagen).

### Cell growth and apoptosis assay

Murine MIN6 β cell and Raw 264.7 macrophage cell lines were cultured in DMEM containing 10% FBS and 1% penicillin-streptomycin and maintained at 37 °C in a 5% CO_2_ atmosphere. Cells were seeded one day prior to being treated with varying concentrations of recombinant platelet basic protein (R&D, catalog number 1091-CK-025/CF) for 24 h followed by an additional 24 h cytokine cocktail (100 ng/mL IFN-γ: R&D, catalog number 485-MI-100, 10 ng/mL TNF-α: R&D, catalog number 410-MT-010, and 5 ng/mL IL-1β: R&D, catalog number 401-ML-005) treatment. Apoptosis was measured by caspase-Glo 3/7 assay (Promega, catalog number G8092), according to the manufacturer’s instructions.

### Exosome isolation and detection from human plasma

Deidentified human plasma was purchased from BioIVT. To avoid the components contained in plasma from interfering with the detection of exosomes, the plasma was purified by size exclusion chromatography (70 nm qEVsingle, IZON) to separate plasma components from exosomes. The fractions containing exosomes were incubated overnight on ExoView® chips (NanoView Biosciences) pre-coated with capture antibodies against human CD9, CD63, CD81, and negative control IgG. To detect proteins carried by exosomes, the chips were further incubated with fluorescently (CF488A)-labeled anti-CXCL7 antibody (Biorbyt, orb667781). The processed chips were scanned in the ExoView® instrument (NanoView Biosciences), and the results were analyzed with the NanoView analysis software.

## Results

### Meta-analysis and data processing

Plasma EV proteomics datasets were searched in the main public repositories for untargeted proteomics associated with the ProteomeXchange consortium, MassIVE, and PRIDE, identifying 24 potential studies. To keep the datasets consistent and allow to perform a single quantification method, only label-free data collected by data-dependent acquisition in orbitrap mass spectrometers were used in the study. This allowed us to use the intensity-based absolute quantification (iBAQ) method and normalize the datasets by relative copy number, enabling the comparison across different experiments. We also removed studies that were from the analysis of post-translational modifications (phosphorylation and glycosylation), that had no raw data files, insufficient metadata details and no biological replicates. One additional study was removed based on the low coverage. A total of 7 studies were eligible for the meta-analysis (see flow diagram for inclusion/exclusion criteria Fig. S1). To further make the meta-analysis consistent, we excluded samples associated with disease, and only the 105 mass spectrometry datasets from control samples were used (see characteristics in Table 1 with number of samples and purification method) (Fig. 1a). The data were processed with MaxQuant for peptide/protein identification and quantification, leading to the identification of 4,611 proteins (Table S1). A principal component analysis showed that the samples cluster based on the separation method (Fig. S2), supporting the idea that each method provides a different profile. We performed clustering analysis to group proteins based on their abundance distribution. To have discrete groups, proteins were clustered by the k-means method. The ideal number of clusters were tested in increments of 5 from 10 to 25. We evaluated the ideal number of clusters by performing an enrichment analysis of the top 100 EV proteins from the Vesiclepedia database (Table S1) and considered the ideal number of clusters to be the ones that provided a cluster with the best enrichment of the top 100 EV proteins. A total of 15 clusters were found to exhibit the best enrichment of the top 100 EV proteins (Fig. 1b, Table 2). Among the 15 clusters, clusters 10 and 11 were significantly enriched with the top 100 EV proteins from Vesiclepedia (1.3- and 1.7-fold, respectively; p-values of 0.02583 and 0.00139, respectively).

**Table 2:** Enrichment of the top 100 extracellular vesicle proteins from Vesiclepedia across the different protein clusters in the proteomics meta-analysis.

Cluster	Top 100	Proteins in cluster	Fold enrichment	*p*-value	Representative proteins
1	12	777	0.6	0.01608	PPIA, NEDD8, ENO1
2	3	191	0.6	0.15103	H2AC4, CST2, Immunoglobulins
3	4	144	1.1	0.1976	KNG1, VWF, CLEC3B, HBB, FCN3
4	0	99	0.0	0.07808	APOC1, APOA2, APOA4, APOD, APOA1
5	2	90	0.9	0.27153	ALB, TF, SERPINA8, F12, F10, F2, A1BG
6	3	70	1.7	0.15905	FGG, F9, C7, C8A, C8B, C9, IGKC
7	0	52	0.0	0.26385	FGB, FBA, CFH
8	1	49	0.8	0.36434	Immunoglobulins
9	25	1132	0.9	0.06748	Ribosomes, RNA-binding proteins, tRNA synthases
10	32	997	1.3	0.02583	CD63, CD81, ESCRT proteins, RAB proteins, integrins
11	30	720	1.7	0.00139	CD9, CD40, HLA, RAB proteins, integrins, N-glycosylation proteins
12	2	92	0.9	0.26967	A2M, C3, C5, F13B, C1QA, C1QB, Immunoglobulins
13	0	72	0.0	0.1574	APOB, APOC3, APOC4, APOE, APOF, APOM
14	1	33	1.2	0.36889	HPR, Immunoglobulins
15	1	93	0.4	0.22365	APOC1, APOD, LPA

As the Vesiclepedia top 100 proteins were selected based on how often they appear in the literature, it is likely that contaminants that are commonly associated with EVs are included in that list^22^. Therefore, we performed a systematic review of the literature to obtain an improved list of human EV proteins that were validated by immunogold electron microscopy, a gold-standard technique to validate the localization of proteins in EVs. The literature search resulted in 204 papers that we examined manually and 48 matched our inclusion criteria, as shown in Fig. S3. A total of 47 EV proteins validated by immunogold electron microscopy were identified (Table S2). From this list, 28 identified were also found in our proteomics meta-analysis, of which 20 were grouped into clusters 10 and 11 (Fig. 1c), representing a 71.4% true-positive rate with a significant enrichment of 1.9-fold (Fisher’s exact test p-value = 0.00018917). Overall, the clustering analysis was effective in enriching for EV proteins.

### Contaminants

**Fig. 2:**
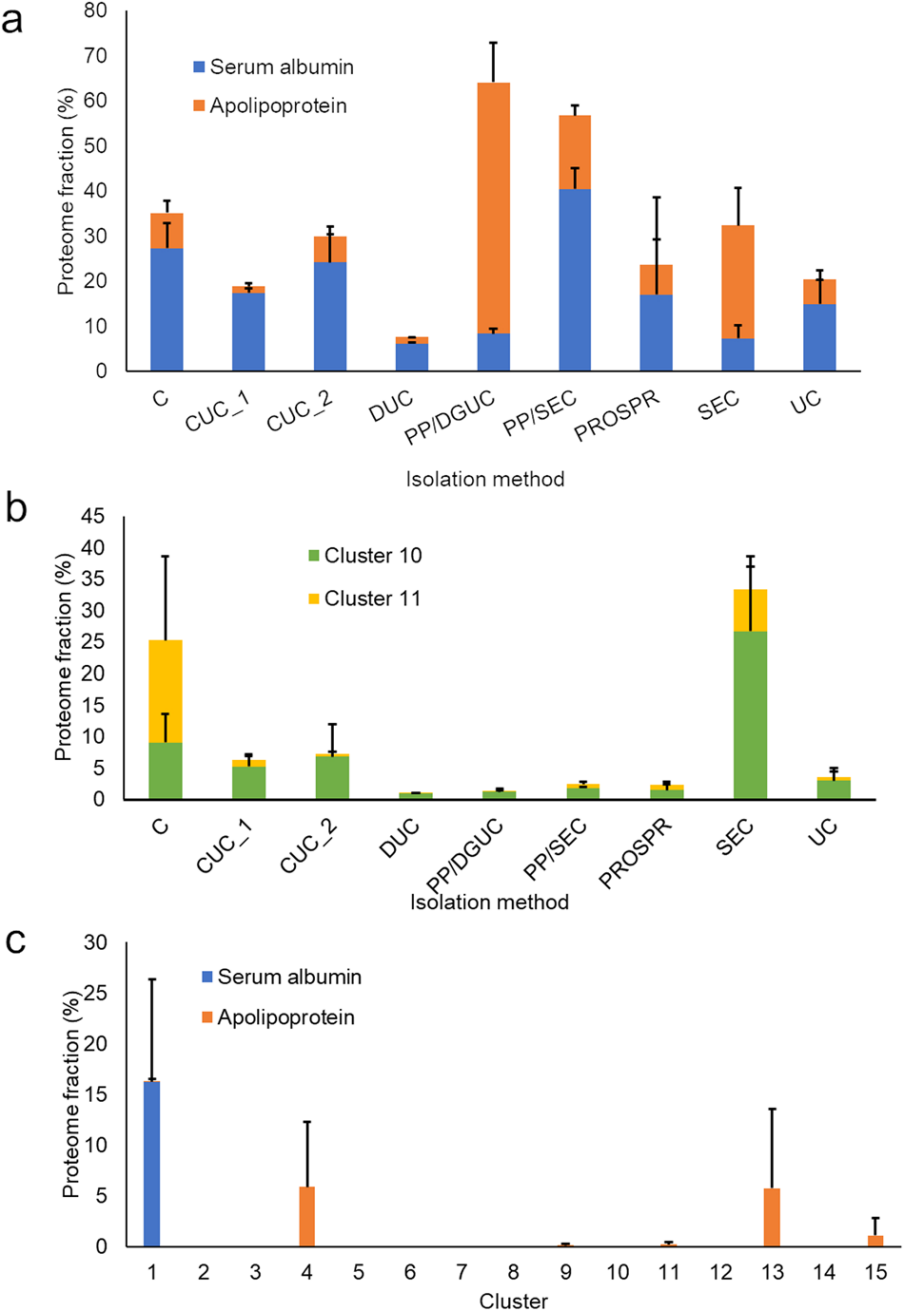
Relative abundance of proteins across different extracellular vesicle purification methods and clusters of the proteome meta-analysis. The proteome samples were quantified by intensity-based absolute quantification (iBAQ) and each protein was normalized to the total sample amount. (**a**) Abundances of common extracellular vesicle preparation contaminants across different purification methods. (**b**) Abundances across different purification methods of clusters 10 and 11, which are enriched in EV markers. (**c**) Abundances of common extracellular vesicle preparation contaminants across different clusters of the proteome meta-analysis. Abbreviations: C - centrifugation, CUC - cushion ultracentrifugation, DGUC - density gradient ultracentrifugation, DUC - dilution followed by ultracentrifugation, PP - polymer-based precipitation, PROSPR - PRotein Organic Solvent PRecipitation, SEC - size-exclusion chromatography, UC - ultracentrifugation.

We examined the profile of well-known contaminants (human serum albumin, lipoproteins - based on the apolipoprotein subunits) of plasma EV preparations to better understand the performance of different methods. For methods based on centrifugation (centrifugation - C, cushion ultracentrifugation - CUC, dilution followed by ultracentrifugation - DUC and ultracentrifugation - UC), 10% to 35% of the total proteome consisted of proteins were human serum albumin and lipoproteins (Fig. 2a). Compared to the UC, the additional step of CUC failed to reduce the proportion of contaminants in the samples, or to increase the proportion of EV proteins in clusters 10 and 11 (Fig. 2b). The best recovery of proteins from cluster 11 was achieved with C only (Fig. 2b). Protein Organic Solvent PRecipitation (PROSPR) had a similar contamination profile compared to the centrifugation, but with lower recovery of clusters 10 and 11. Polyethylene glycol polymer precipitation (PP) followed by density gradient ultracentrifugation (DGUC) highly enriched for lipoproteins, with apolipoprotein subunits accounting for over 50% of the total protein abundance in the sample (Fig. 2a). When PP was followed by size-exclusion chromatography (SEC), a similar proportion of contaminants was observed, but the serum albumin was the major contaminant (Fig. 2a). SEC alone had approximately 35% of the sample comprised of the 3 analyzed contaminants, predominantly lipoproteins (Fig. 2a). However, SEC alone yielded the best recovery of cluster 10 and the second highest for cluster 11, with these 2 clusters representing approximately 35% of the sample proteome combined (Fig. 2b).

We examined the profile of serum albumin and lipoproteins across 15 clusters. Clusters 10 and 11, which showed the best enrichment in EV proteins, had a low amount of these contaminants in contrast to cluster 1 that showed a high amount of albumin, over 15% (Fig. 2c). Clusters 4, 13, and 15 were abundant in apolipoproteins, ranging from 1.5% to 6.5% (Fig. 2c). These results showed that clusters 10 and 11 were enriched in EV proteins and depleted of common plasma contaminants.

### Markers of plasma EVs

**Fig. 3:**
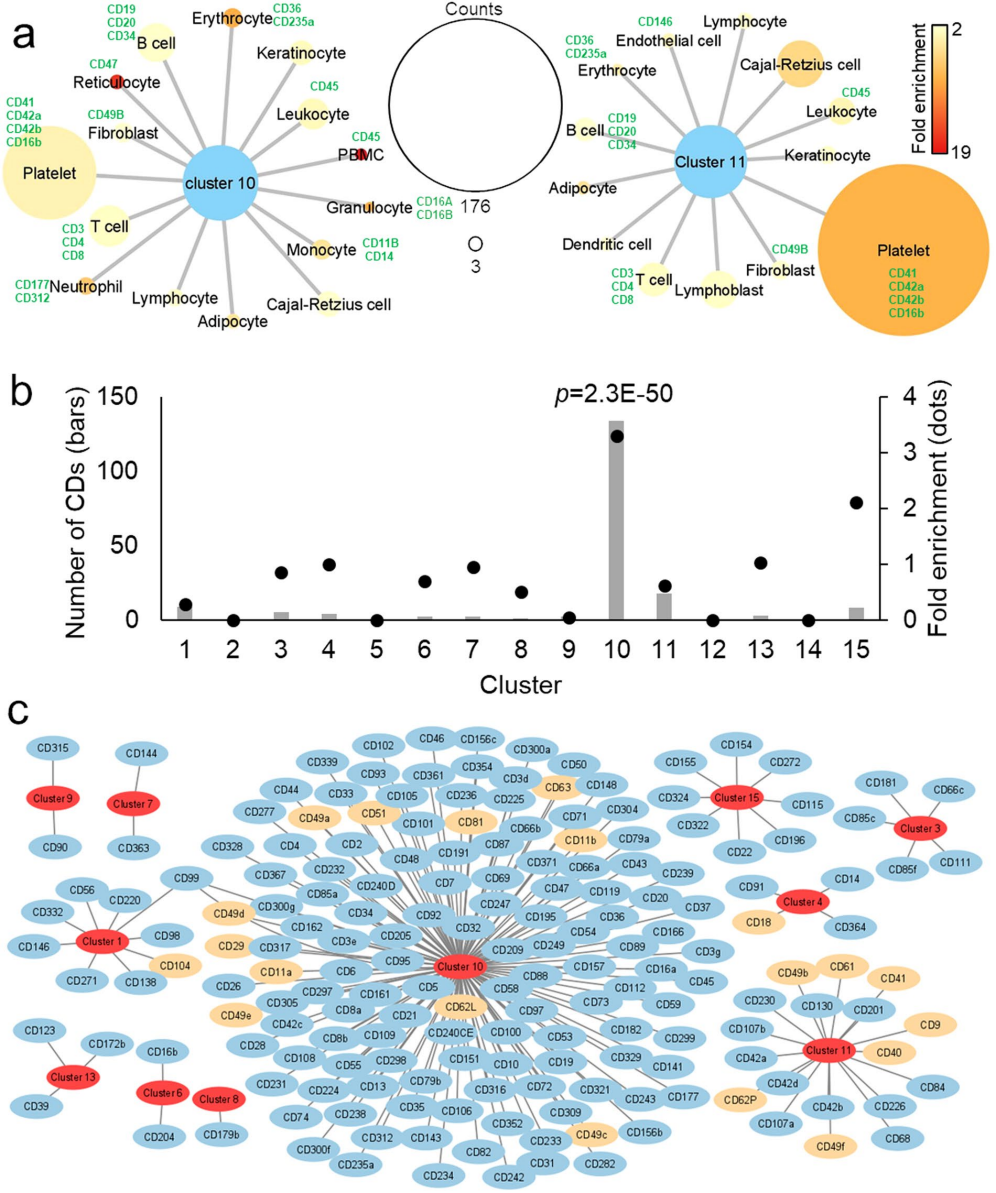
Cellular markers. (**a**) Cells enriched in proteins from clusters 10 and 11 in the DAVID analysis. Node (circle) sizes represents number of proteins and colors, the fold enrichment. (**b**) Number of CD antigens and enrichment (compared to the total identified proteins) across different clusters. Significance was determined by Fisher’s exact test. (**c**) Network of CD antigens across different clusters. Classical extracellular vesicle markers are colored in orange.

To study cellular markers present in plasma EVs, we performed a cell-enrichment analysis using Database for Annotation, Visualization, and Integrated Discovery (DAVID). The analysis was based on the UniProt Knowledgebase tissue database. It showed that clusters 10 and 11 were overrepresented in proteins from blood and immune cells, including platelets, erythrocytes, T cells, B cells, monocytes, and dendritic cells (Fig. 3a). The clusters were also enriched in proteins from fibroblasts, keratinocytes, adipocytes, and Cajal-Retzius cells, a type of cortical neurons (Fig. 3a). To look for proteins that can be used as markers of specific cells, we examined the cluster of differentiation (CD) antigens, which are cell surface proteins used to distinguish cell populations by immunoassays. We found that CD antigens were enriched in cluster 10 while depleted in the other clusters. Out of 188 total CD antigens identified, 133 cell-specific CD antigens were present in cluster 10 (Fig. 3b,c, Table S3). These included markers of specific cells, such as CD45 (leukocyte, PBMC), CD3 (T cell), CD4 (T-helper cell), CD8 (cytotoxic T cell), CD19, CD20 and CD34 (B cell), CD14 and CD11b (monocyte), CD47 (reticulocyte), CD16a and CD16b (granulocyte), CD177 and CD312 (neutrophil), CD36 and CD235a (erythrocyte), CD41, CD42a, CD42b and CD16 (platelet), and CD146 (endothelial cells) (Fig. 3a). These results show that the plasma EVs carries dozens of CD markers that can be further used for immunology and cell biology experiments.

### Pathways enriched in EV proteins

**Fig. 4:**
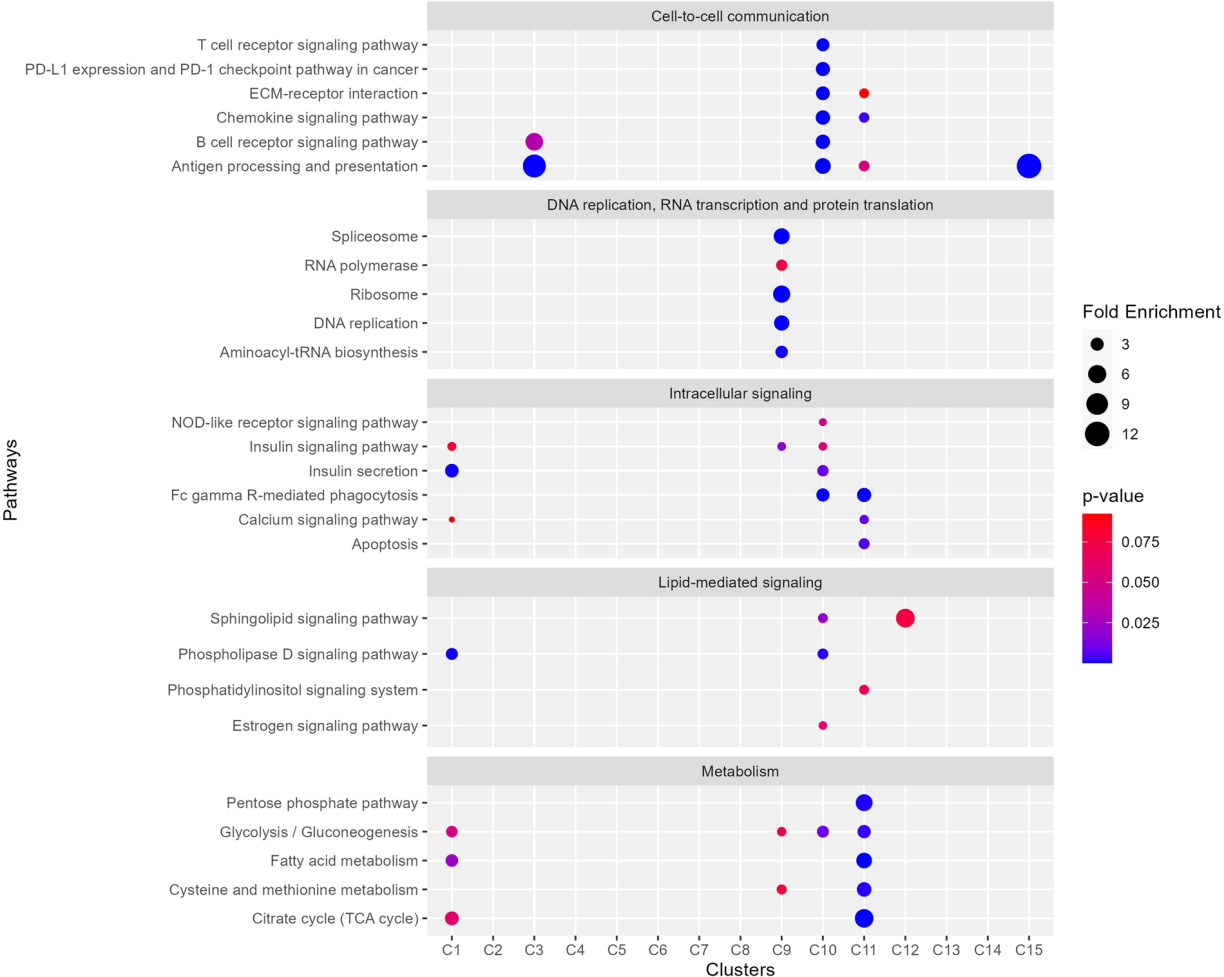
Pathway-enrichment analysis. Distribution of selected pathway enrichment across different clusters. The circle sizes represent the fold enrichment and the colors, p-values.

To investigate possible functions of plasma EVs, we performed a functional-enrichment analysis using the DAVID tool and the KEGG annotation (the complete list of enriched pathways is available in Open Science Framework^28^). Clusters 10 and 11 were enriched in proteins from several cell-to-cell communication pathways, such as pathways on antigen processing and presentation, ECM-receptor interaction, PD-L1 expression and PD-1 checkpoint, B-cell receptor signaling, chemokine signaling, and T-cell receptor signaling (Fig. 4). These clusters were also enriched in proteins from other cell signaling pathways, such as apoptosis, calcium signaling, Fc γ receptor-mediated phagocytosis, insulin signaling, insulin secretion pathway, and NOD-like receptor signaling pathways (Fig. 4). Clusters 10 and 11 were also overrepresented in lipid (estrogen, phosphatidylinositol, phospholipase D, and sphingolipid)-mediated signaling pathways (Fig. 4). Metabolic proteins, such as from the citrate cycle, cysteine and methionine metabolism, fatty acid metabolism, glycolysis/gluconeogenesis, and pentose phosphate pathways, were enriched, especially in cluster 11 (Fig. 4). Proteins from DNA replication, RNA transcription (RNA polymerase and spliceosome), and protein translation (ribosome and aminoacyl-tRNA biosynthesis) processes were enriched in cluster 9 (Fig. 4), which was not enriched with EV markers (Table 2). These results suggest that EVs may play a role in diverse communication and signaling pathways in addition to being enriched with specific metabolic pathways.

### Plasma EV proteins as potential type 1 diabetes biomarkers

**Fig. 5:**
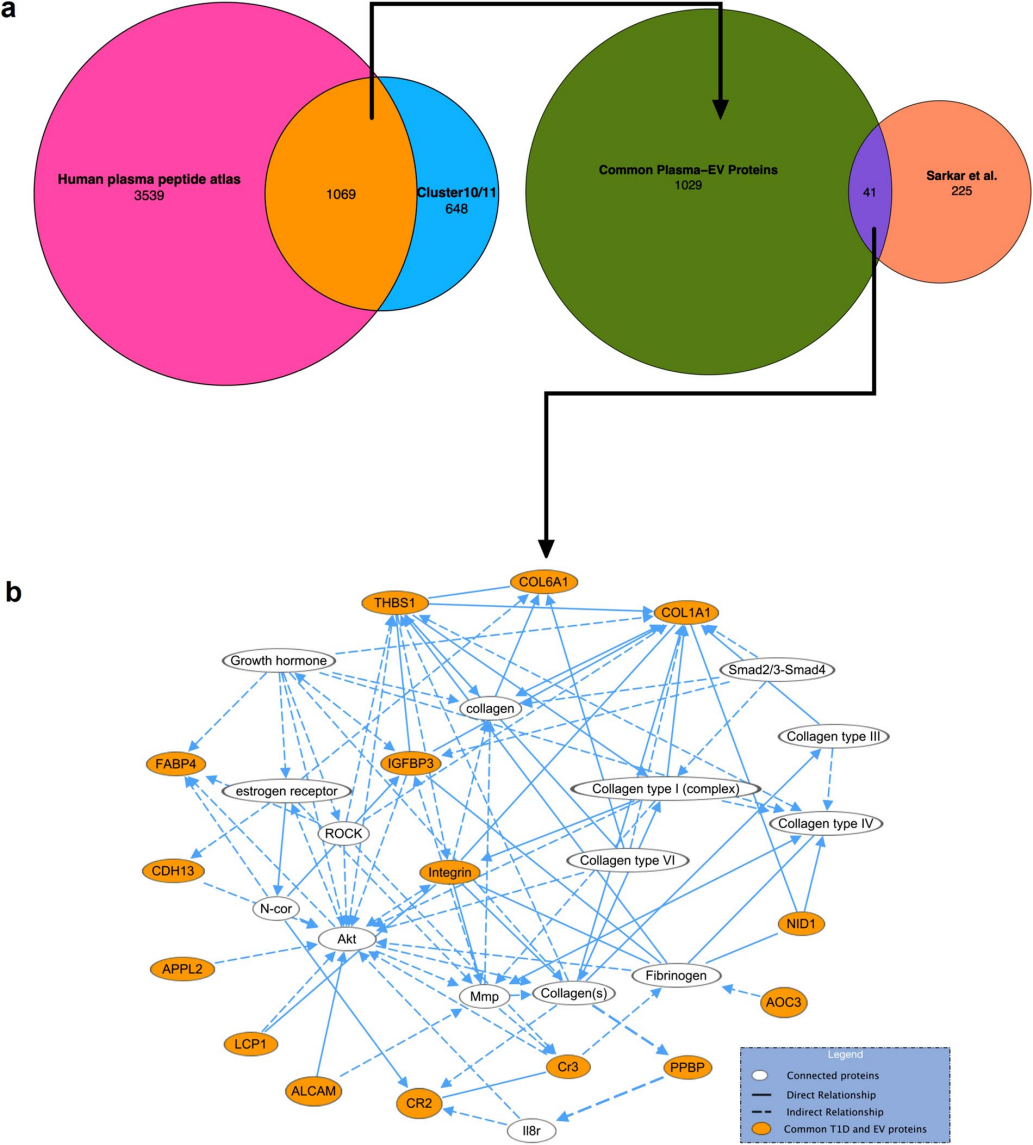
Evaluation of extracellular vesicle proteins potential as biomarker candidates. (**a**) Extracellular vesicle (EV) proteins from clusters 10 and 11 were compared the Human Plasma PeptideAtlas database, and a systematic review of type 1 diabetes (T1D) biomarker candidates^30^. (**b**) Network of T1D biomarker candidates present in EVs. The network was built after pathway-enrichment analysis performed with Ingenuity software (Qiagen).

We next evaluated the potential of plasma EV proteins as biomarker candidates. For this, we compared our data with the human plasma proteins deposited in PeptideAtlas (4608 proteins). PeptideAtlas is an invaluable resource containing not only peptides from proteins identified in plasma, but also information on peptides with established assays for targeted proteomics, a main technique used for biomarker analyses^29^. By matching against the 1717 proteins from clusters 10 and 11 with the PeptideAtlas, we found 1069 common proteins (Fig. 5a, Table S4). Therefore, selecting targets among these 1069 proteins yields a higher chance of generating successful targeted proteomics assays. To further explore the potential of these proteins as biomarkers, we compared the 1069 proteins with a list of 266 T1D biomarker candidates from a systematic review that we recently conducted^30^. This analysis resulted in a list of 41 plasma EV proteins that are also T1D biomarker candidates (Fig. 5a, Table S5). As changes in proteins that make them biomarkers are often drivers or consequences of the pathological process of the diseases, we took a closer look at the 41 plasma EV proteins that are T1D biomarker candidates. We performed an Ingenuity pathway analysis (Qiagen), which revealed an enrichment in proteins from cytokine/chemokine signaling (Fig. 5b). These results showed that plasma EVs carries several proteins that are candidates for T1D biomarkers, with a good likelihood to be developed in targeted proteomics assays and possible contributions to the disease process.

### Validation and biological activity of an EV protein identified in our meta-analysis

**Fig. 6:**
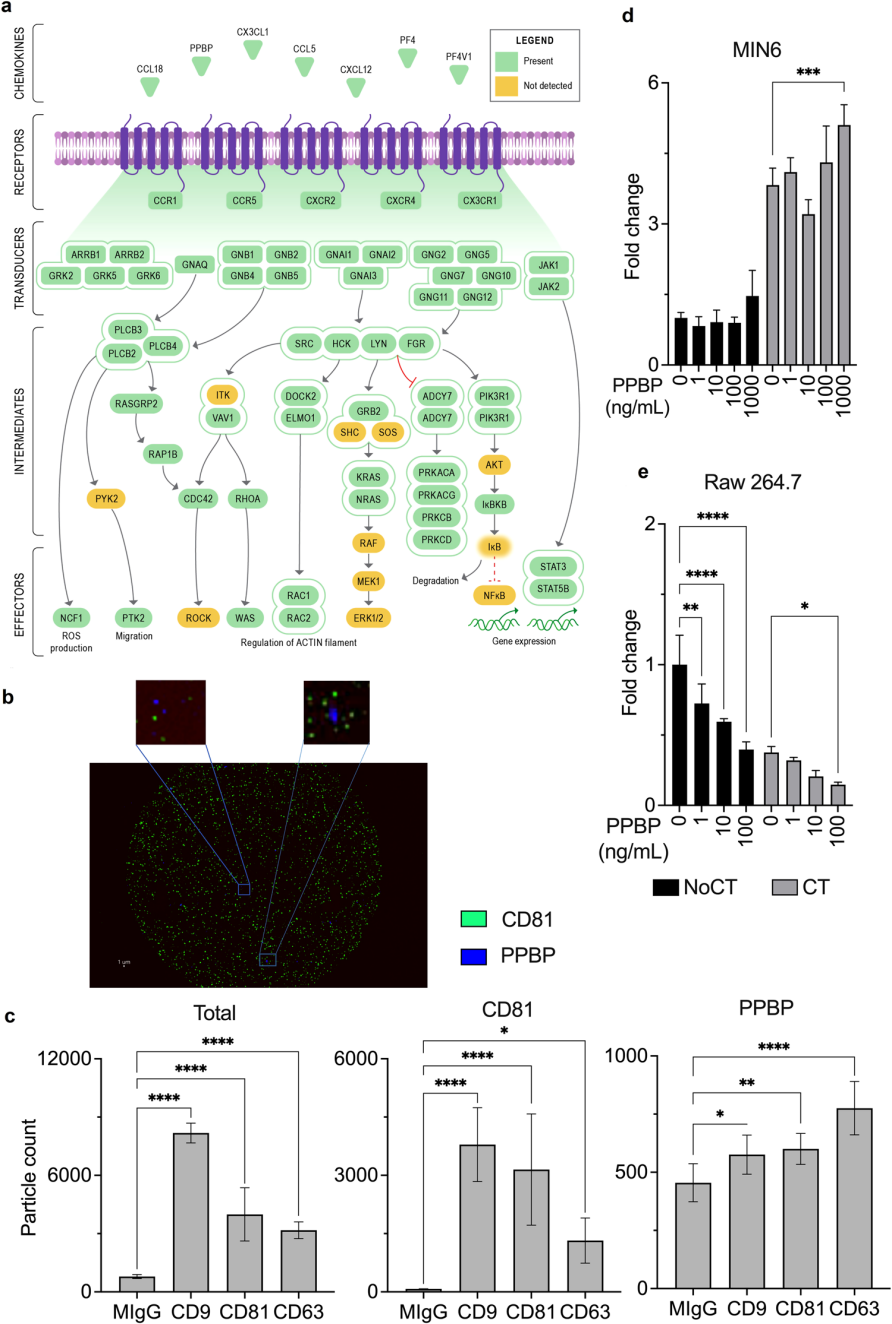
Chemokine signaling components in extracellular vesicles and their activity in β cells and macrophages. (**a**) Chemokine signaling pathway components detected in plasma extracellular vesicles (clusters 10 and 11). (**b**) Showing the representative ExoView image of extracellular vesicles captured from human plasma in the chip and stained for CD81 and proplatelet basic protein (PPBP). (**c**) The bar graph represents number of total extracellular vesicles captured in the ExoView chip, and subpopulations containing CD81 and PPBP (n = 3). Significance by 1-way ANOVA *p ≤ 0.05, **p ≤ 0.01, & ****p ≤ 0.0001 (**d,e**) The bar graphs represent fold change in apoptosis in Raw 264.7 and MIN6 cells pretreated with different concentrations of PPBP for 24 h and then treated with a cytokine cocktail (CT, 100 ng/mL IFN-γ, 10 ng/mL TNF-α, and 5 ng/mL IL-1β) vs. control (noCT). N = 4 ± SD, Significance by 2-way ANOVA *p ≤ 0.05, **p ≤ 0.01, ***p ≤ 0.001 & ****p ≤ 0.0001.

As the chemokine-signaling pathway was highly enriched in clusters 10 and 11 and among the plasma EV proteins that are T1D biomarker candidates (Figs. 4, 5b), we decided to further investigate this pathway. We were especially interested in this pathway as many chemokines play a role in T1D development^31^. A detailed annotation of this pathway revealed a total of 7 chemokines and 5 receptors detected, along with complete signaling branches related to the JAK/STAT and SRC/DOCK2/RAC1-2 cascades (Fig. 6a). Among the identified EV-chemokines, proplatelet basic protein (PPBP, also known as chemokine CXCL7) (Figs. 5b, 6a) has been validated as a T1D biomarker, with reports of its elevated levels in the plasma prior to and at the onset of the disease^32,33^. One of PPBP’s receptors, CXCR2, was also present in the plasma EVs (Fig. 6a). Therefore, we hypothesize that PPBP might have a role in T1D development. We first validated the presence of PPBP in EVs using the ExoView technology. In this technology, EVs are captured in microchips with antibodies against CD9, CD63, and CD81 then specific EV proteins are imaged by immunostaining using a fluorescence microscope. As a positive control, we stained for CD81 and our target PPBP. The images show an example of EVs captured with anti-CD9 antibodies and stained for CD81 and PPBP. The results show that the majority of the captured EVs contained CD81, while only a small fraction contained PPBP (Fig. 6b). The quantification showed that despite the number of particles positive for PPBP being low in plasma, it is significantly higher than the negative control for EV capture with pre-immune mouse antibody (MIgG), confirming the presence of this protein in plasma EVs (Fig. 6c). We next sought to determine if this increased level of plasma PPBP has a role in regulating cytokine-mediated apoptosis, a key process during T1D development. We pre-treated MIN6 β cells and Raw 264.7 macrophages with recombinant PPBP, prior to exposure to a cytokine cocktail (interferon γ, interleukin β, and tumor necrosis factor α). We found that PPBP induces MIN6 apoptosis, but in contrast, PPBP reduces Raw 264.7 apoptosis (Fig. 6d,e). Overall, these results show a remarkable enrichment of the chemokine-signaling pathway in EVs. In addition, we validated the presence of PPBP in EVs, which was identified in our meta-analysis. Furthermore, PPBP might play a role in T1D development by differentially regulating apoptosis in β cells and macrophages, two cell types that play key roles in T1D development.

## Discussion

Obtaining highly pure EV preparations is a complex task. For instance, plasma lipoproteins have similar sizes, and some particles have similar densities as EVs, making it difficult to attain pure EVs with techniques based on size and density^6–8^. Another challenge is the unavoidable presence of a high abundance of plasma proteins, such as albumin. To address this challenge, multiple purification steps with albumin depletion columns have been explored^34^. However, multi-step purifications lead to substantial sample losses, with almost complete EV loss after 3 purification steps^6^. Therefore, there is a need to better understand the characteristics of different isolation methods so that optimal approaches can be incorporated in each study. By applying a meta-analysis to datasets refined the composition of plasma EVs, leading two clusters with 1717 proteins enriched in EV proteins. We validated these clusters by performing a systematic review of human EV proteins that were analyzed by immunogold electron microscopy. Our analysis also quantified differences in contaminants across these methods and, herein, we provide insights into strengths and weaknesses of each method.

Centrifugation-based purification methods had similar contamination profiles. Diluting the plasma before the centrifugation seems to have a strong contaminant-reducing effect, through diminishing the concentration of proteins and consequently, their aggregation or absorption to EVs. However, the larger volume in DUC also affected the recovery of EVs during centrifugation. Inclusion of purification steps based on the same physical properties (e.g., ultracentrifugation vs. cushion ultracentrifugation) provided no added benefit in sample purity. Sequential purification steps had better results when using different physical-chemical properties, such as SEC followed by DGUC, but they led to a low EV recovery (~1%)^11^. Polymer-based precipitation (PP) had large amounts of lipoprotein contamination, probably due to their mechanism of enriching for EVs by binding to lipids. These findings suggest that understanding of contaminant characteristics is key to understanding limitations of each isolation technique, ways to improve sample preparation quality, and ability to distinguish activities of EVs from those of contaminants. In terms of EV sample recovery, high speed centrifugations had a low recovery, probably due to aggregation or disruption of EVs^35,36^. SEC had the best performance recovering proteins from clusters 10 and 11 combined, which is consistent with previous observation that SEC can recover 35% of the plasma EVs^11^. Centrifugation at 10,000xg showed an enrichment to cluster 11, probably by enriching for EVs with higher density. Therefore, for applications where pure materials are not required, such as targeted proteomics analysis, a single-step enrichment might be more appropriate than attempts to obtain highly purified materials in trace amounts.

Cell-specific proteins were identified in EVs from a variety of immune cells including lymphocytes, monocytes, neutrophils, and dendritic cells. These were accompanied by the presence of cell-surface CD antigens, which can be used as cellular markers. Many of these CD antigens have roles in cell signaling and cell-to-cell communication, consistent with earlier reports of EV participation in those events. A classic example of intercell communication by EVs is antigen presentation and T-cell activation by B-cell derived EVs^37^. Our pathway analysis further showed that plasma extracellular vesicles are also enriched in cell-to-cell communication and signaling proteins. However, the extent of the enrichment and the completeness of some signaling pathways were unprecedented. For instance, we found that the EV clusters had 133 CD antigens and complete signaling pathways. This opens the possibility that EVs can not only be a signaling messenger but complement cells that lack specific signaling pathways.

As a proof of principle for how these data can be applied, we also investigated the possibility of targeting EV proteins as T1D biomarkers and found that 41 current T1D biomarker candidates are present in EVs. Among these proteins is PPBP, which has been shown to be a T1D biomarker candidate in two separate studies^32,33^. We found that PPBP regulates apoptosis of β cells and macrophages. However, its role in T1D development is still poorly understood. The protection of macrophages and induction of β-cell apoptosis might represent a signal amplification mechanism, leading to an increase in β-cell death. The PPBP cleavage product, CXCL7, is a major chemokine that primes neutrophil migration by aggregating with platelets and inducing the formation of neutrophil extracellular traps (NETs)^38^. Neutrophil-platelet aggregates have been found to be abundant in blood of pre-diabetic and recent T1D onset mice and humans^39^. In addition, NETs were shown to cause β-cell death *in vitro*^39^, further supporting that PPBP might have a role in T1D development. However, additional work is needed to have a mechanistic understanding on the action of PPBP in T1D.

Overall, we provided an alternative approach for characterizing the EV composition. We found that C and SEC methods led to the best plasma EV recovery, but none of the studied methods led to pure preparations. Our analysis showed that plasma EVs are derived from a variety of cells and that they are enriched in cell surface markers and cell-to-cell communication molecules. Our study provides lists of proteins that are highly likely to be bona fide EV proteins than can be used for prioritization for mechanistic and biomarker studies. As an example, we validated the presence of PPBP in EVs and further showed that controls apoptosis in culture β cells and macrophages and might have a role in T1D development.

### Supplementary information

Supplemental figures

Supplemental Tables

## Data Availability

Additional data are available in Open Science Framework^28^. This includes MaxQuant results and parameter files for each dataset under the folder “MaxQuant_results_and_parameters”, which are named based on their Pride accession numbers; an excel file containing the complete abundance data matrix under the folder “Processed_data_matrix”; and the results from the DAVID functional enrichment analysis under the folder “Enrichment analyses”.

## Code availability

The R and Python scripts written to generate Fig. 5 and Figure S2 are available in GitHub^23^.
