# Supplementary material for: A proteomic meta-analysis refinement of plasma extracellular vesicles
Source: Sci Data. 2023 Nov 28;10:837. doi: 10.1038/s41597-023-02748-1 (PMC10684639; doi:10.1038/s41597-023-02748-1)
Supplement: Supplementary file 1 — Supplemental figures [file 41597_2023_2748_MOESM1_ESM.docx]

***Supplemental Figures***

**A proteomic meta-analysis refinement of plasma extracellular vesicles**

Milene C. Vallejo^1^, Soumyadeep Sarkar^2^, Emily C. Elliott^2^, Hayden R. Henry^2^, Samantha Powell^2^, Ivo Diaz Ludovico^2^, Youngki You^2^, Fei Huang^3^, Samuel H. Payne^1^, Sasanka Ramanadham^4^, Emily K. Sims^5^, Thomas O. Metz^2^, Raghavendra G. Mirmira^3^, Ernesto S. Nakayasu^2^*

^1^Department of Biology, Brigham Young University, Provo, UT, USA.

^2^Biological Sciences Division, Pacific Northwest National Laboratory, Richland, WA, USA.

^3^Department of Medicine, The University of Chicago, Chicago, IL, USA.

^4^Department of Cell, Developmental, and Integrative Biology, and Comprehensive Diabetes Center, University of Alabama at Birmingham, Birmingham, AL, USA.

^5^Department of Pediatrics, Center for Diabetes and Metabolic Diseases, Indiana University School of Medicine, Indianapolis, IN, USA.

Correspondence: Ernesto S. Nakayasu, Biological Sciences Division, Pacific Northwest National Laboratory, Richland, 99354, WA, USA. Email: ernesto.nakayasu@pnnl.gov

**Identification of studies**

**Included**

**Identification**

Studies identified from:

Data repositories (n = 2)

Studies (n = 24)

(Keywords used “extracellular vesicles”, “exosomes”, “microvesicles”, AND “plasma”, “serum”)

Date: Nov-Dec/2021

Studies excluded:

Reason 1: no healthy controls (n = 6)

Reason 2: labelled proteomics (n = 3)

Reason 3: glycoprotein (n = 1)

Reason 4: phosphorylation (n= 1)

Reason 5: no raw data provided (n= 2)

Reason 6: insufficient metadata provided (n=2)

Reason 7: no biological replicates (n=1)

Studies screened

(n = 24)

**Screening**

Reports assessed for data quality

(n = 8)

Studies excluded:

Reason 1: poor quality/low coverage (<200 identified proteins) (n= 1)

Studies included in review

(n = 7)

**Fig. S1 –** Workflow and inclusion/exclusion criteria of studies for the proteomics meta-analysis. Studies were searched in the ProteomeXchange-associated data repositories Pride and MassIVE, and included/excluded with the criteria listed in the figure.


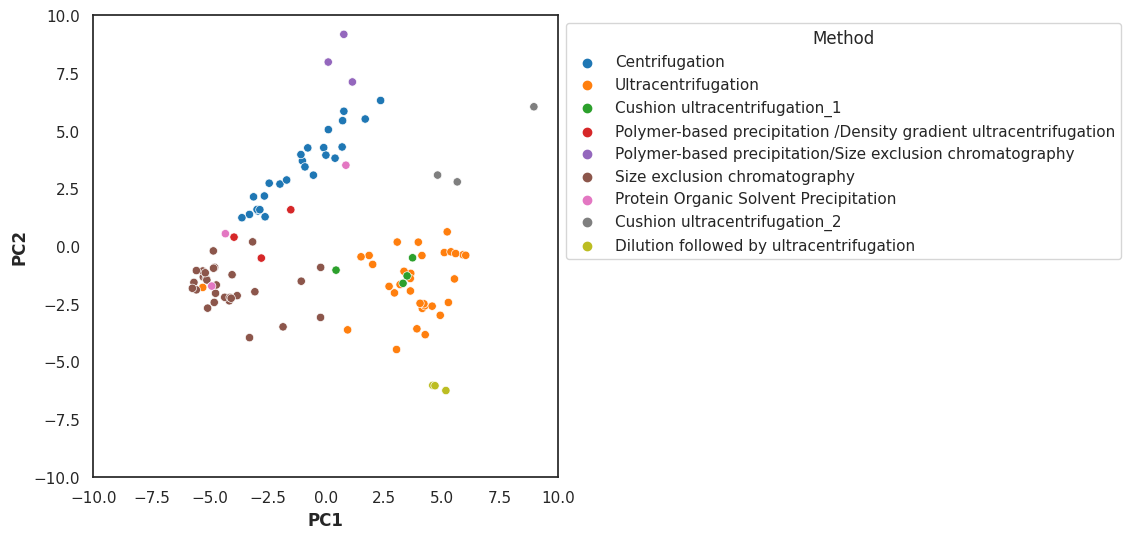


**Fig. S2. Principal component analysis of the proteomics meta-analysis data**. Visualization of variance in the methods used for EV-extraction and identification of patterns and relationships among proteins.

**Identification of studies**

**Screening**

**Included**

**Identification**

Studies identified from PubMed:​ (n = 204)​

(Keywords used “Extracellular vesicles”, “EV” “Microvesicles” “MV” “Exosome” AND “Immunogold” AND “human”)​

Date: 6/28/2023

Studies removed before screening:

Duplicate studies between searches (n = 88)

Studies included in review

(n = 48)

Studies assessed for eligibility​

(n = 109)​

Date: (7/28/2023)

Studies screened

(n = 116)

Studies excluded:

Studies with no full text or with no access (n = 7 )​

Studies excluded:

Model systems other than human (n = 25)​

Not performed in EVs (n = 19)​

Did not identify proteins (n = 5)​

Did not use immunogold (n = 2)​

**Fig. S3 –** Workflow and inclusion/exclusion criteria of studies for the systematic review of validated extracellular vesicle proteins. Studies were searched in the PubMed and included/excluded with the criteria listed in the figure.
